# Examining the longitudinal associations between activity limitations, instrumental supports and social participation in osteoarthritis: A CLSA population-based study

**DOI:** 10.1371/journal.pone.0299894

**Published:** 2024-03-27

**Authors:** Anthony V. Perruccio, Calvin Yip, J. Denise Power, Mayilee Canizares, Elizabeth M. Badley

**Affiliations:** 1 Schroeder Arthritis Institute, University Health Network, Toronto, Canada; 2 Arthritis Community Research and Epidemiology Unit, Toronto, Canada; 3 Institute of Health Policy, Management and Evaluation, Dalla Lana School of Public Health, University of Toronto, Toronto, Canada; 4 Department of Surgery, University of Toronto, Toronto, Canada; 5 Dalla Lana School of Public Health, University of Toronto, Toronto, Canada; Zarqa University, JORDAN

## Abstract

**Objective:**

In osteoarthritis (OA) research, disability is largely studied within the context of activities of daily living. Broader consequences for social participation are often overlooked. In prior work, instrumental supports received and their perceived availability were shown to play a role in the maintenance of social participation. Two indicators of social participation were identified, diversity and intensity. The current study extends the findings from this prior cross-sectional work by examining these relationships longitudinally.

**Methods:**

Data are from the baseline and 3-year follow-up questionnaires of the Canadian Longitudinal Study on Aging, a population-based study of people ages 45–85 years at baseline. The sample was restricted to those who at baseline reported a doctor diagnosis of OA (n = 4104). Using structural equation modeling, latent variables were derived at each time point for activity limitations, instrumental supports perceived and received, and social participation diversity and intensity. Longitudinal factorial invariance was assessed. Model covariates included age, sex, education, income, marital status, smoking status, obesity, and number of chronic conditions.

**Results:**

For all latent variables, strong factorial longitudinal invariance was found. Activity limitations increased over time. Greater baseline social participation intensity was associated with increases in later intensity and diversity. Increasing activity limitations were associated with decreases in social participation and with increasing receipt of instrumental supports; they were not associated with changes in perceived availability of supports. However, increasing perceived availability was positively associated with social participation intensity.

**Conclusions:**

With a goal of increasing social participation, findings suggest a focus on interventions to reduce activity limitations in OA is necessary. Findings additionally highlight an important role for perceived availability of instrumental supports in maintaining or improving social participation in OA, in addition to current social participation, particularly intensity, for future social participation status.

## Introduction

Longer life can be accompanied by increased risks of age-related, nonfatal, and disabling conditions. Particularly for conditions that can develop in early or middle adulthood, this may also mean more years lived with disability [1]. A number of nations have emphasized ‘healthy aging’ as a population health priority [2–4]. Healthy aging has been defined as a process of creating and enhancing opportunities to maintain and improve health, independence, and quality of life, with the benefits for older individuals including reduced time to recovery from illness, reduced risk of disease onset, and improved overall personal, family and community well-being. [5] Osteoarthritis (OA) plays a particularly important role within this context of increased risks owing to its high prevalence in the population (between 22–38% among adults aged 50+), increasing prevalence with increasing age, its key role as a cause of pain and disability, and the fact that there is no known OA cure or disease-modifying therapies [6–9].

While social participation has been defined in several ways, a recent review reported that many definitions focus on one’s participation in activities that offer interactions with others in community and collective spaces. [10] Social participation has been described as an important component of healthy aging [11–14], particularly for those with disabilities, and reports of its benefits for life quality and satisfaction and health have been published [13,15–20]. Yet, despite OA’s high prevalence and significant negative impacts on individuals and the broader population, relatively limited work has evaluated the association between OA and social participation. The current work aims to improve our understanding by identifying factors associated with changes in social participation among individuals with OA.

Prior work [21], underpinned by the World Health Organization International Classification of Functioning, Disability, & Health (ICF) framework [4,22,23], identified factors across varied domains that were associated with OA and social participation. Initially in this prior work in OA, a single social participation construct was posited. Two latent indicators ultimately emerged, one related to *diversity* in social participation (‘SP-Diversity’), described as reflecting the range or variety of activities undertaken on a regular basis, the other to *intensity* of social participation (‘SP-Intensity’), described as reflecting more the degree or extent of participation [21]. Findings from confirmatory factor analysis work supported the distinct nature of these latent indicators, which included a low correlation between them, *r =* 0.23, as did findings of unique associations with a number of covariates [21]. The study found that the magnitude of the negative association between activity limitations in OA and social participation was diminished when instrumental supports (e.g. help with personal care, housework, transportation) were considered. There were two domains of instrumental supports, *received* instrumental supports (supports which respondents indicated they received) and *perceived* instrumental supports (supports which respondents indicated they believed would be available to them should they need them). Received instrumental supports had a role only for social participation diversity, while perceived availability of instrumental supports had a role for social participation intensity. While these were interesting findings, a major limitation of the work was its cross-sectional nature, and there has been limited work, particularly in OA, to understand whether these might contribute to changes in social participation. As the ultimate goal is targeted efforts to facilitate greater social participation to promote healthy aging in OA, understanding whether changes in these factors may contribute to changing social participation or overcoming barriers to social participation is a vital step towards identifying possible avenues of intervention. [24,25] The present work contributes to filling this knowledge gap.

In this longitudinal population-based study we sought to extend the cross-sectional findings to determine, among individuals with OA, if changes in activity limitations and instrumental supports influence changes in social participation.

## Methods

### Sample

The Canadian Longitudinal Study on Aging (CLSA) is a population-based cohort study of Canadians aged 45 to 85 years. The study collects data on a broad set of factors, including sociodemographic characteristics, psychosocial-, behavioural-, and health-related characteristics. [26] The cohort has been designed to follow participants for 20 years or until death. The present study used baseline (cycle 1) and 3-year follow-up (cycle 2) data from the CLSA Tracking Cohort, corresponding with beginning and ending recruitment dates of September 22, 2011 to May 3, 2014 and December 7, 2015 to December 18, 2018, respectively. The cohort is comprised of participants selected randomly from each of the 10 Canadian provinces, and participants completed questionnaires administered via computer assisted telephone interview. This present study focused specifically on those individuals who at baseline reported that a doctor had told them they had osteoarthritis in the knee, hip, or hand (n = 4258). Written informed consent was obtained from all CLSA participants prior to data collection. The current study received ethics approval from the University Health Network Research Ethics Board (#16–5883.4), and the data were first accessed for research purposes on August 5, 2021.

### Study variables

The main analysis, later described, assesses relationships among latent variables within a structural equation modeling framework. Here we describe the composition of these latent variables, linking individual items to latent variables.

*Social participation* (baseline and follow-up). Respondents were asked a range of discretionary social involvement questions which tapped into social participation, with some variation in question format. The first asked about trips/activities “you typically make in a week”, with respondents indicating yes/no to each, the second queried whether specific statements related to an individual’s participation were applicable, with yes/no responses, and the third covered frequency of several community-related activities, with response options “never”, “at least once a year”, “at least once a month”, “at least once a week”, or “at least once a day”. Two latent social participation factors were developed, labelled *SP-diversity* and *SP-intensity*, corresponding with findings from previous work (latent variable derivations are described below) [21]. Social participation factors with their corresponding items are provided in the supplemental file (S1 Table in S1 File).

*Activity limitations* (baseline and follow-up): Respondents were queried about experiencing difficulty performing specific activities (e.g. reaching; kneeling; standing; etc.; see S2 Table in S1 File), with no/yes/unable to do/don’t do on doctor’s orders response options. For each ‘yes’ response, the degree of difficulty (a little, somewhat, very) was elicited, and overall difficulty for each activity was characterized as none, a little, somewhat, very, or unable to do. ‘Don’t do on doctor’s orders’ was included in ‘unable to do’. A latent activity limitations factor was derived.

*Instrumental Supports* (baseline and follow-up): For each of four scenarios, respondents indicated the frequency with which they had available supports if they should need them (none of the time, a little, some, most, all the time). The four scenarios included a) having someone to help if they were confined to a bed, b) needing help with daily chores if they were sick, c) needing help to prepare meals if they were unable to, and d) needing to be taken to the doctor if needed. From these, an availability of instrumental supports latent factor was developed, labeled **IS-Perceived.** Individuals also indicated if they had received instrumental support in the previous 12-month period due to limitations or a health condition for a) personal care, b) medical/nursing care, c) managing care, d) indoor/outdoor housework, e) transportation, and e) meal preparation/delivery. From these, an instrumental supports latent factor was developed, labeled *IS-Received*.

#### Contextual factors

A number of baseline contextual factors were considered in the present study. These included age, sex, level of education, household income, marital status, obesity (body mass index (BMI; kg/m^2^) ≥30 (obese) or <30 (non-obese)), smoking status, and summed number of self-reported doctor diagnoses (cancer, neurological, mental, respiratory, cardiovascular, ocular, musculoskeletal (excluding OA), and endocrine). In addition to baseline sum, a variable was derived representing the change in number of conditions since baseline. As there were 6.7% and 12.6% missing data for income and education, we retained a ‘missing’ category for these variables.

### Statistical analysis

Baseline sociodemographic and health-related characteristics of the sample are provided for those with and without follow-up, along with baseline and follow-up status proportions based on the individual response items corresponding with the latent factors described above.

A structural equation modelling framework was used for the study. The main focus of our conceptual model concerned the relationships among the latent variables at follow-up. Adjustment was made for the effect of respective baseline status on each latent variable. Thus the follow-up latent variables reflect change within the analysis. A simplified version of our conceptual model is depicted in S1 Fig in S1 File. Corresponding with confirmatory factor analysis findings in previous work [21], latent factors were derived in the present study for *SP-Diversity*, *SP-Intensity*, *Activity Limitations*, *IS-Perceived* and *IS-Received* based on the items noted above. As the individual item responses were ordered categorical in nature, the weighted least squares means and variance adjusted (WLSMV) estimator was used. Furthermore, as the analysis consisted of repeated latent variables over time, testing for longitudinal configural and simultaneous metric and scalar (metric+scalar) measurement invariance was undertaken. This process assessed whether the constructs (e.g. activity limitations) were measured equally at the two time points within the group, ensuring that changes observed over time could be attributed to actual change in the construct under investigation rather than change in the measurement properties of the construct. Overall good model fit was defined by values of Root Mean Squared Error of Approximation (RMSEA)≤0.06, Standardized Root Mean Square Residual (SRMR)≤0.08 and Comparative Fit Index (CFI)>0.90, while longitudinal measurement invariance was established with CFI and RMSEA change values of ≥-0.002 and ≤0.007, respectively, between the metric+scalar invariant model and the configural model, and by examination of local fit indices. [27–29] The chi-square fit statistic was not considered owing to its sensitivity to large sample size.

Following assessments for longitudinal measurement invariance, the compositions of the latent variables at follow-up were found to be consistent with findings for the baseline latent variables. Evidence of strong longitudinal measurement invariance was found, with only 1 of 38 items displaying non-invariance, each latent variable set had good overall fit for the configurally invariant model, and differences in fit between configurally invariant and metric+scalar invariant models were below noted thresholds (S3 Table in S1 File).

With longitudinal measurement invariance established for each set of latent variables, all latent factors were brought together into a single analysis. Based on findings from prior work, baseline activity limitations were specified as predictors of future instrumental supports and social participation, and baseline instrumental supports as predictors of future social participation. In addition, each baseline social participation factor was specified as a predictor of both future social participation factors. To mitigate potential baseline confounding (e.g. those with high baseline SP-intensity exhibiting fewer activity limitations, or fewer comorbidities, etc.), all baseline latent variables were permitted to correlate, and contextual factors were included as predictors of the latent variables. This final comprehensive model displayed good overall fit, with RMSEA = 0.019 (90% C.I.: 0.018, 0.019), P(RMSEA≤0.05) = 1.000, CFI = 0.952, TLI = 0.950 and SRMR = 0.058. The distinction between the two social participation latent variables at follow-up was confirmed, evident from their low correlation (*r* = 0.307). Analyses were performed using Mplus V.8.0, and p-values < 0.05 were deemed statistically significant.

## Results

Of 4258 eligible individuals with OA at baseline, 88 were missing some baseline contextual data and 66 provided no follow-up data. The final analytical sample consisted of 4104 individuals (95% of all eligible). No differences in baseline status were found between those with and without follow-up data (Table 1). At baseline, the overall sample had a mean age of 66 years, 61% were female, and 32.4% were obese.

**Table 1:** Baseline sample characteristics of those with OA.

	Sample with follow-up data (n = 4192) (mean or % (n))	Sample with missing follow-up (n = 66) (mean or % (n))	p-value*
Mean age (years) (SD)	66.13 (9.9)	64.83 (10.7)	0.316
Female	61.4% (2574)	60.6% (40)	0.895
Level of Education			
≤ High school	25.6% (1075)	28.8% (19)	0.788
Post-secondary	61.7% (2587)	60.6% (40)	
Missing	12.6% (530)	10.6% (7)	
Household Income			
≤ $49,999	38.7% (1622)	42.4% (28)	0.908
$50,000 to $99,999	35.0% (1466)	31.8% (21)	
$100,000+	19.6% (821)	18.2% (12)	
Missing	6.8% (283)	7.6% (5)	
Obese	32.4% (1360)	34.8% (23)	0.464
Missing	0.5% (20)	1.5% (1)	
Current smoker	8.5% (355)	6.1% (4)	0.280
Missing	0.4% (16)	1.5% (1)	
Married/Common Law	64.6% (2709)	65.1% (43)	0.929
Missing	0.0% (0)	0.0% (0)	

*T-test or chi-square test, as appropriate.

The mean number of chronic conditions increased from 2.5 at baseline to 3.0 at follow-up (Table 2). To convey overall changes in the sample, summary results are presented in Table 2 for activity limitations, instrumental supports and social participation overall (i.e. by category of latent factor), rather than for 39 individual items. Forty percent of the sample reported activity limitations in 3+ activities at baseline, increasing to 45% 3 years later. While there was a small non-significant increase overall in the proportion perceiving having available supports, to all items, there was a significant increase in the proportion reporting have received supports by follow-up, 24% having received support with at least one item at follow-up compared to 19% at baseline (Table 2). Overall, while the diversity of social participation increased over the 3-year period, the overall intensity of social participation decreased in this OA sample (Table 2). Considering the intensity items, the proportion engaging in at least 3 of the 5 specific items decreased from 89% to 82%, and the proportion reporting being engaged at least once a month in at least 4 of the 6 community activities decreased from 43% to 38% (Table 2).

**Table 2:** Baseline and follow-up data for individuals with OA (analytical sample n = 4104).

	Baseline (mean or %)	Follow-up (mean or %)	Paired test p-value
Mean chronic condition count (SD)	2.50 (1.7)	2.97 (1.7)	<0.001
Activity limitations (13 items)			<0.001
1+ difficulties	74.7%	76.4%	
2+ difficulties	54.9%	58.6%	
3+ difficulties	40.4%	45.2%	
Instrumental support—perceived availability (4 items)			0.513
‘none of the time’ to all items	0.6%	0.7%	
‘all of the time’ to all items	32.9%	34.1%	
Instrumental support—received (6 items)			<0.001
1+ ‘yes’ responses	18.6%	23.9%	
2+ ‘yes’ responses	11.6%	15.1%	
3+ ‘yes’ responses	7.3%	8.4%	
Social participation			
Diversity (4 items)			
Undertakings typically made in a week			<0.001
‘no’ to all 4 items	29.1%	9.5%	
‘yes’ to at least 3 of 4 items	33.6%	45.3%	
‘yes’ to all 4 items	15.9%	19.7%	
Intensity (11 items)			
Individual			<0.001
‘no’ to all 5 items	0.5%	0.7%	
‘yes’ to at least 3 of 5 items	89.2%	82.1%	
‘yes’ to all 5 items	31.1%	25.7%	
Community-related			<0.001
‘never/yearly’ to all 6 items	4.1%	6.4%	
‘at least once a month’ for at least 4 of 6 items	43.3%	38.7%	
‘at least once a month’ for all 6 items	8.1%	5.9%	

As expected, the baseline level of each latent factor was positively and significantly associated with its respective status at follow-up. For example, greater activity limitations at baseline were associated with greater activity limitations by follow-up. For these repeated effects, greater magnitudes were found for activity limitations, IS-perceived and SP-intensity, with standardized regression coefficients (95% CL) of 0.74 (0.71, 0.77), 0.58 (0.55, 0.61), and 0.83 (0.79, 0.87), respectively. Smaller repeated effects were found for IS-received and SP-diversity, 0.18 (0.11, 0.25) and 0.09 (0.04, 0.14), respectively. Baseline SP-diversity had minimal effect on future SP-intensity (stand. coeff. = 0.04 (0.01, 0.08)), while baseline SP-intensity had a much larger effect on future SP-diversity (stand. coeff. = 0.58 (0.52, 0.65)).

The associations amongst the follow-up latent factors within the final comprehensive model are shown in Table 3. Increases in activity limitations were associated with significant increases in reports of having received instrumental supports, and significant and similar decreases in both SP-diversity and SP-intensity. While increases in IS-received did not appear to be associated with changes in social participation, increases in perceived availability of supports if needed were positively and significantly associated with increases in SP-intensity over the 3 years.

**Table 3:** Standardized regression coefficients between primary study variables^†^.

	Dependent Latent Variables			
	Increase in Instrumental Supports		Increase in Social Participation	
Predictor Variable	IS-Received	IS-Perceived Availability	SP-Diversity	SP-Intensity
	Standardized regression coefficients (95% CL)			
Increases in Activity Limitations	**0.56** **(0.46, 0.67)**	0.04 (-0.04, 0.13)	**-0.27** **(-0.39, -0.15)**	**-0.29** **(-0.38, -0.19)**
Increase in IS-Received			0.04 (-0.04, 0.11)	0.02 (-0.03, 0.08)
Increase in IS-Perceived Availability			0.05 (-0.01, 0.12)	**0.16** **(0.10, 0.21)**

^†^Statistically significant (p<0.05) estimates are bolded; model adjusted for prior statuses and sociodemographic and health-related contextual factors.

The associations between the sociodemographic and behavioural/health-related contextual factors and the main study variables, from the same comprehensive model as above, are presented in Table 4. The behavioural and health-related factors were all associated with increasing activity limitations (e.g. obesity and comorbidity were associated with increases in activity limitations), while none of these were directly associated with social participation. A greater number of comorbid conditions was associated with increases in received instrumental supports and decreases in perceived availability of supports if needed. Finally, individuals from households with lower income were more likely to report decreases in perceived availability of supports compared to those with higher household income, and being married/common law was associated with increases in perceived availability of supports.

**Table 4:** Standardized regression coefficients (±standard error) between sociodemographic and health-related contextual factors and primary study variables.

	Dependent Variables				
	Increases in Activity Limitations	Increases in Instrumental Support		Increases in Social Participation	
Predictor		IS-Received	IS-Perceived Availability	SP-Diversity	SP-Intensity
	Standardized regression coefficient (95% CL) *				
Sociodemographic					
Age	0.10^*^ (0.07, 0.13)	0.05^*^ (0.001, 0.09)	0.02 (-0.01, 0.06)	-0.07^*^ (-0.12, -0.02)	-0.05^*^ (-0.08, -0.02)
Female (vs. male)	-0.00 (-0.03, 0.03)	0.04 (-0.01, 0.08)	0.02 (-0.02, 0.05)	-0.05^*^ (-0.09, -0.01)	0.02 (-0.01, 0.05)
Household Income					
Low vs. high	0.03 (-0.01, 0.08)	-0.01 (-0.08, 0.06)	-0.08^*^ (-0.13, -0.04)	0.04 (-0.02, 0.11)	0.04 (-0.01, 0.09)
Middle vs. high	0.02 (-0.02, 0.06)	-0.01 (-0.05, 0.08)	-0.05^*^ (-0.09, -0.01)	0.03 (-0.03, 0.09)	0.02 (-0.03, 0.06)
Missing	0.03 (-0.00, 0.06)	-0.01 (-0.06, 0.02)	-0.03 (-0.06, 0.01)	0.05^*^ (0.01, 0.10)	-0.01 (-0.04, 0.03)
Education					
≤secondary vs. post-secondary	-0.00 (-0.03, 0.02)	0.01 (-0.03, 0.05)	0.01 (-0.03, 0.04)	0.05^*^ (0.01, 0.09)	-0.03 (-0.06, 0.00)
Missing	0.01 (-0.02, 0.03)	-0.02 (-0.07, 0.02)	0.01 (-0.02, 0.04)	0.04 (-0.00, 0.09)	0.00 (-0.03, 0.03)
Married/common law (vs. not)	-0.02 (-0.05, 0.01)	0.02 (-0.03, 0.06)	0.09^*^ (0.05, 0.12)	-0.04 (-0.09, 0.01)	0.01 (-0.02, 0.05)
Behavioural and health					
Obese (vs. not)	0.07^*^ (0.04, 0.10)	-0.01 (-0.05, 0.04)	-0.02 (-0.05, 0.01)	0.02 (-0.02, 0.06)	0.03^*^ (0.01, 0.06)
Current Smoker (yes vs. no)	0.03^*^ (0.00, 0.06)	0.01 (-0.03, 0.06)	-0.04^*^ (-0.07, -0.01)	0.02 (-0.03, 0.07)	-0.01 (-0.04, 0.02)
Initial comorbidity count	0.10^*^ (0.07, 0.13)	0.06^*^ (0.01, 0.11)	-0.05^*^ (-0.08, -0.01)	0.01 (-0.04, 0.06)	-0.00 (-0.04, 0.03)
Comorbidity increase	0.17^*^ (0.14, 0.20)	0.05^*^ (0.01, 0.09)	-0.01 (-0.05, 0.03)	0.03 (-0.02, 0.07)	0.02 (-0.02, 0.06)

*Statistically significant (p<0.05) estimates are bolded. Estimates are from the comprehensive, fully adjusted model.

## Discussion

The study sought to determine if changes in activity limitations and instrumental supports influenced changes in social participation among individuals with OA. In a large, longitudinal population-based sample, we found that increasing activity limitations were associated with decreases in social participation among individuals with OA. Prior intensity of social participation appears to have a greater role than prior diversity in increasing both future intensity and diversity of social participation. Future intensity of social participation was also increased among those perceiving a greater availability of instrumental support should they need them.

Pain and functional limitations are major sequelae of OA, with significant impacts on people’s lives, particularly increased activity limitations, loss of dexterity, and mobility issues often leading to negative social consequences. Even so, it has been suggested that there is a role for social relations in such outcomes. [30,31] Our findings point to the availability of social support as an important factor in improving social participation in people with OA, even against a backdrop of increasing activity limitations with consequent negative impacts on social participation. In a population-based study of social participation among older adults, Xin and Li used functional disability, cognitive impairment, chronic diseases and self-rated health data to derive latent ‘health risk’ groups (labeled low, moderate and high health-risk) [32]. They found that with the increases in health risks, the contributions of community support and psychological resources to older adults’ social participation decreased. In contrast, only the contribution of social network support (availability of emotional and instrumental support) to social participation increased. This suggests that instrumental social support may play a key role in social participation for individuals with chronic diseases and functional limitations [32].

Physical activity is recommended as a key symptom and disease management strategy in OA care, though OA-related activity and mobility limitations may be perceived as barriers to such uptake. Even within this context, social support can play an important role. Previous studies have reported positive associations between individual social support and physical activity levels. [33,34] In a Swedish population-based study, Chen et al. reported that among older adults higher levels of social support and positive affect were strongly associated with less daily time spent sitting and more engagement and time spent in light physical activity. [35] There may be reciprocal effects between social participation and health [36], such that better physical activity and health allow for greater social participation, and in turn improved activity, health, and social support and participation. [37] Different strategies have been recommended to incorporate physical activity into healthcare settings, including physical activity counselling by healthcare providers, and written prescriptions for behavioural change and follow-up strategies, for example. [38] The current work suggests that the availability of social supports, or perceptions of their availability, may also be an important factor for care providers to raise with patients and their family members in discussions around physical activity and self-management, particularly as people with OA often report worry about needing help from others. [39] Health professionals can also help individuals with identifying relevant resources or programs and promoting connectedness with their community; social support interventions in community settings have been identified as effective in increasing levels of physical activity [40].

Baseline SP-intensity was more strongly associated with increases in SP-intensity and SP-diversity than baseline SP-diversity. We also found in this sample of 45–85 year olds with OA, older age to be associated with decreases in social participation intensity and less so diversity, as well as with more received instrumental supports. This may suggest differences in the significance of each of diversity and intensity in social participation over the lifespan and possibly over increasing severity of OA over time. [41] Holt-Lunstad argues that while social support (actual or perceived availability) is often assumed to be more relevant to health and social connection in older age, emerging evidence suggests that it is relevant across life stages, with some data indicating that younger age groups could be at equal or greater risk of loss. [42] This is particularly salient in OA, where work has shown that various impacts of OA in younger adult age groups can be equal to or greater than the impact among older age groups [43,44].

Time since OA diagnosis was not available for consideration in the current study. Interestingly, it has been reported that among adults with chronic conditions, greater disease duration may be related to greater participation, for some, possibly attributed to adaptive approaches or coping mechanisms developed over time. [45] Furthermore, in this same study, greater perceived social support was linked with a better capacity to participate socially. Importantly, as characterized, the latter corresponded more so with participation intensity in the current work, and thus supports our findings connecting social participation intensity with instrumental support availability. While we imagine that receiving instrumental supports might lead to increases in social participation, we did not find this to be the case. It may be that for those achieving a higher degree of disease and symptom severity which may trigger their request for and receipt of support, the received support may not necessarily lead to increases in social participation but may help delay or prevent reductions. OA symptom and disease severity were not available for this population-based sample.

Neighbourhood factors can affect social participation but were not considered in this study. [46,47] Different research perspectives specify that aspects of a person’s placement in social relations and context (e.g. frequency of social interactions, social support, social cohesion) operate both on the individual and the neighborhood level. [48] At the individual level, it is understood to affect health status through one’s use of their social resources, such as individual social support. At the neighbourhood level, it has been suggested that perceived social capital can have some positive effect, though this has been minimal in many cases and inconsistent, on individual health indicators. [49,50] In a community-based study, Lagaert et al. evaluated the effect of individual and neighborhood social capital on individual self-rated health from a multilevel perspective, arguing that previous work in the area had not adequately captured or analytically accounted for neighbourhood level measures. [48] They found social capital operated significantly more at the individual level than the neighborhood level, and highlighted primarily the importance of individual social capital, particularly social support, as the estimated neighborhood-level social factors were either non-significant or had a significantly smaller effect on self-rated health. Given known relationships between self-rated health and social participation, [51–53] this suggests that even with the inclusion of neighbourhood effects in the present study, it is unlikely they would have altered our conclusions, though may have identified possible additional pathways to influence social participation. With OA and ageing, the social and physical environment can become progressively more restrictive, potentially altering the nature, extent or space within which social participation occurs. [54,55] Considering this along with the varied definitions of social participation in the literature,[10] we acknowledge the boundaries of the perspectives in the present study.

Doctor-diagnosed OA was self‐reported, and there is the potential therefore for recall and reporting bias. Also, the CLSA only captured OA at the hand, knee and hip. While OA is most frequent as these sites, this nevertheless may limit generalizability. We did not focus on the specific joint affected. While the activity limitation questions include some activities where the lower joints are likely predominantly used, and others where the hand and upper joints are likely predominantly used, the reality is that in many cases several joint sites are simultaneously involved to some degree. Furthermore, some of the social activities may involve, for example, the upper extremity joints, but this may be the case more so for some individuals than others (e.g. variability in hobbies across individuals). The separating of joint sites, activity limitations and social activities to understand the independent effect of specific joint sites would require several assumptions. In addition, and importantly, the reality often overlooked in OA is that many have multiple joints involved, [56–60] so that the ‘separating’ of joints can be artificial. Nevertheless, to distinguish impacts by joint site is of interest, particularly from an intervention stand point. This would require further study, however, with a more comprehensive assessment of OA, including all joints, and with activities specifically selected to be joint-specific.

This was an observational study that identified factors associated with changes in social participation. A next step will be to determine if intervening to optimize these factors has its intended effects on social participation, a focus for future work.

## Conclusions

Improved social participation is an emerging key target in efforts to promote healthy aging, and this is particularly relevant for conditions such as OA where there are limited treatment strategies that can directly influence the disease course. OA is a threat to healthy aging and to the health systems intended to support and care for an aging society. This study, based on a large, prospective and population-based sample of individuals with OA, largely confirms cross-sectional study results, and shows that for individuals with OA, interventions targeted at activity limitations are important, as these limitations have detrimental effects on social participation, but the work draws additional attention to the important role of available instrumental supports in maintaining or improving social participation. Improving social supports may be a key factor for increasing the likelihood that individuals experience healthy aging, and efforts to spotlight the need, availability and benefits of social supports and social participation in clinical and public health settings should be encouraged.

## Supporting information

S1 File (DOCX)
