# Correction: Examining the longitudinal associations between activity limitations, instrumental supports and social participation in osteoarthritis: A CLSA population-based study

**DOI:** 10.1371/journal.pone.0329802

**Published:** 2025-08-05

**Authors:** Anthony V. Perruccio, Calvin Yip, J. Denise Power, Mayilee Canizares, Elizabeth M. Badley

The following information is missing from the Funding statement: AV Perruccio is supported by an award from Arthritis Society Canada STAR-20-0000000012. The source had no involvement in study design, analysis, interpretation, writing, or decision to submit for publication.
