# Supplementary material for: Examining the longitudinal associations between activity limitations, instrumental supports and social participation in osteoarthritis: A CLSA population-based study
Source: PLoS One. 2024 Mar 27;19(3):e0299894. doi: 10.1371/journal.pone.0299894 (PMC10971692; doi:10.1371/journal.pone.0299894)
Supplement: S1 File — (DOCX) [file pone.0299894.s001.docx]

**Table S1.** Questionnaire items assessing social participation.^*^

**Table S2.** Questionnaire items assessing activity limitations and degree of difficulty

**Table S3.** Model fit indices for individual repeated sets of latent factors

**Figure S1.** Conceptual model in assessing relationships among activity limitations, instrumental supports and social participation, including contextual factors.

**Table S1.** Questionnaire items assessing social participation.^*^

| Social Participation-Diversity^†^ | |
| --- | --- |
| 1 | Recreational/leisure shopping, restaurants |
| 2 | Recreational/leisure trips to park, other outdoor spaces |
| 3 | Visiting friends and family |
| 4 | Social activities (e.g. seniors recreational centres) |
|  | |
| Social Participation-Intensity^**^ | |
| Individual | |
| 5 | I have a hobby or pastime |
| 6 | I have taken a holiday in Canada in the last 12 months |
| 7 | I have taken a holiday outside of Canada in the last 12 months |
| 8 | I have gone on a daytrip or outing in the last 12 months |
| 9 | I use the internet and/or e-mail |
|  | Community-related |
| 10 | Family or friendship based activities outside the household |
| 11 | Sports or physical activities that you do with other people |
| 12 | Educational and cultural activities involving other people such as attending courses, concerts, plays, or visiting museums |
| 13 | Neighbourhood, community or professional association activities |
| 14 | Volunteer or charity work |
| 15 | Any other recreational activities involving other people, including hobbies, gardening, poker, bridge, cards, and other games |
|  |  |
| ^*^ Preface statement in questionnaire: “Now some questions about your social activities”  ^†^Question: “What kind of trip(s) do you typically make in a week, whether by car, public transit, walking or other means?” (instruction to interviewer: Code all that apply).  ^**^Question for items 5-9: “Which of these statements apply to you?” (instruction to interviewer: Code all that apply);  Question for items 10-15: “The next questions are about community-related activities that you may have participated in during the past 12 months. In the past 12 months, how often did you participate in…” Response options for each: at least once a day; at least once a week; at least once a month; at least once a year; never | |

**Table S2.** Questionnaire items assessing activity limitations and degree of difficulty

| Question: Do you have any difficulty….(see list below)  Response options: Yes; No; Unable to do; Don’t do on doctor’s orders  For each ‘Yes’ response, respondents were subsequently probed with,  “Would you say that the degree of difficulty is…”  Response options: A little difficult; Somewhat difficult; Very difficult |
| --- |
| List of Activities |
| 1. reaching or extending your arms above your shoulders |
| 1. stooping, crouching, or kneeling down |
| 1. pushing or pulling large objects like a living room chair |
| 1. lifting ten pounds (or 4.5 kg) from the floor, like a heavy bag of groceries |
| 1. standing for a long period, around 15 minutes |
| 1. sitting for a long period, say 1 hour |
| 1. standing up after sitting in a chair |
| 1. walking alone up and down a flight of stairs |
| 1. walking 2 to 3 neighbourhood blocks |
| 1. making a bed |
| 1. washing your back |
| 1. using a knife to cut food |
| 1. with recreational or work activities in which you take some force or impact through your arm, shoulder, or hand (e.g., golf, hammering, tennis, typing, etc.) |

**Table S3.** Model fit indices for individual repeated sets of latent factors

| Repeated latent variable | Model |  | Fit Indices | | | |
| --- | --- | --- | --- | --- | --- | --- |
|  |  |  | RMSEA | CFI | TLI | SRMR |
|  |  |  |  |  |  |  |
| Activity Limitations | Configural Invariance |  | 0.043 | 0.966 | 0.96 | 0.044 |
|  | Metric + Scalar Invariance |  | 0.037 | 0.969 | 0.969 | 0.045 |
|  | difference |  | -0.006 | 0.003 | 0.009 | 0.001 |
|  |  |  |  |  |  |  |
| Instrumental Supports - Perceived | Configural Invariance |  | 0.034 | 0.998 | 0.996 | 0.010 |
|  | Metric + Scalar Invariance |  | 0.022 | 0.998 | 0.998 | 0.010 |
|  | difference |  | -0.012 | 0.000 | 0.002 | 0.000 |
|  |  |  |  |  |  |  |
| Instrumental Supports – Received | Configural Invariance |  | 0.019 | 0.997 | 0.995 | 0.045 |
|  | Metric + Scalar Invariance |  | 0.02 | 0.995 | 0.994 | 0.047 |
|  | difference |  | 0.001 | -0.002 | -0.001 | 0.002 |
|  |  |  |  |  |  |  |
|  |  |  |  |  |  |  |
| Social Participation - Diversity | Configural Invariance |  | 0.019 | 0.998 | 0.995 | 0.02 |
|  | Metric + Scalar Invariance |  | 0.022 | 0.996 | 0.994 | 0.021 |
|  | difference |  | 0.003 | -0.002 | -0.001 | 0.001 |
|  |  |  |  |  |  |  |
| Social Participation - Intensity | Configural Invariance |  | 0.044 | 0.96 | 0.952 | 0.05 |
|  | Metric + Scalar Invariance |  | 0.041 | 0.961 | 0.959 | 0.051 |
|  | difference |  | -0.003 | 0.001 | 0.007 | 0.001 |

**Figure S1.** Conceptual model in assessing relationships among activity limitations, instrumental supports and social participation, including contextual factors.
